# Advanced technologies for future ground-based, laser-interferometric gravitational wave detectors

**DOI:** 10.1080/09500340.2014.920934

**Published:** 2014-06-25

**Authors:** Giles Hammond, Stefan Hild, Matthew Pitkin

**Affiliations:** ^a^ SUPA, School of Physics and Astronomy, University of Glasgow, Glasgow, UK.

**Keywords:** gravitational wave detection, interferometry, thermal noise, quantum noise

## Abstract

We present a review of modern optical techniques being used and developed for the field of gravitational wave detection. We describe the current state-of-the-art of gravitational waves detector technologies with regard to optical layouts, suspensions and test masses. We discuss the dominant sources and noise in each of these subsystems and the developments that will help mitigate them for future generations of detectors. We very briefly summarise some of the novel astrophysics that will be possible with these upgraded detectors.

## 1 Gravitational waves: how to observe the mostviolent events in the Universe?

Over the last century, our ability to view the Universe has undergone many revolutions. Observational astronomy has progressed from a time when the only tool was ground-based visible light photometry to an era of ground-and-space-based multi-wavelength photometry and spectroscopy. These advances have led to countless new discoveries and greatly increased our knowledge of the Universe, but have only been possible through major technological and scientific leaps in instrumentation. Sometimes, this has been through serendipitous applications from different fields, but often it has been the astronomical need that has pushed the technological boundaries.

**Figure 1.**
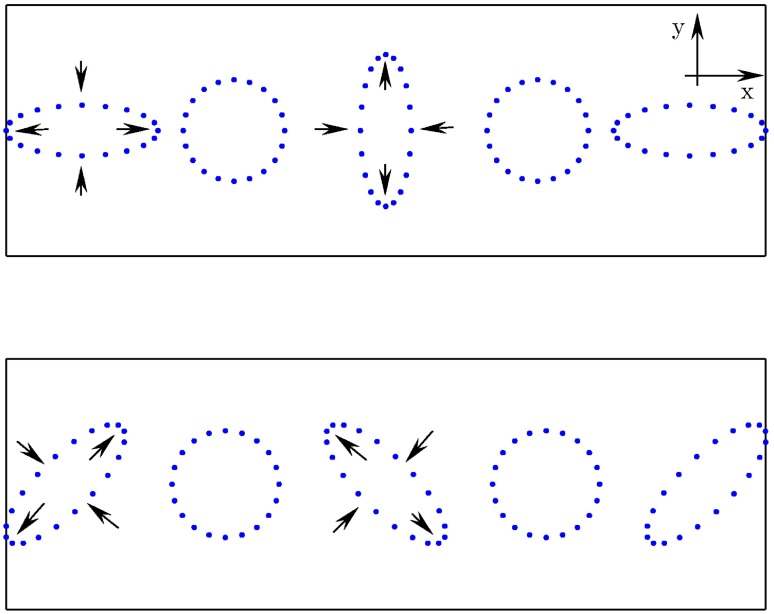
The effect of a passing gravitational wave on a ring of freely falling test particles. The top and bottom panels show the effect of the ‘plus’ and ‘cross’ polarisation modes, respectively. (The colour version of this figure is included in the online version of the journal.)

Gravitational waves (also called gravitational radiation) offer another new window on the Universe moving beyond the electromagnetic spectrum. They are a direct prediction of Einstein’s General Theory of Relativity (GR), although their main properties can be inferred using Newtonian gravity and the principal of special relativity that information (including the influence of gravity) cannot propagate faster than the speed of light [1]. As they travel through space, gravitational waves manifest as a transverse varying tidal strain, which would have the effect of altering the proper distance between freely falling objects. Gravitational waves are quadrupolar in nature and have two polarisation states (called ‘plus’ [+] and ‘cross’ [×]) rotated by 45${}^{\circ }$ with respect to each other (see Figure 1). These states have orthogonal polarisation.

Gravity is the weakest of the fundamental forces and correspondingly the effect of gravitational waves is tiny. Their strength is often measured as a strain in space, where for our ring of particles in Figure 1 a maximum displacement of Δl, given an un-displaced radius of l, means a strain of h=Δl/l. For a source at a distance r, the approximate strain amplitude is given by

(1) $$\begin{array}{c} h\approx \frac{2GM}{c^{2}r}{\left(\frac{v}{c}\right)}^{2}, \end{array}$$

where v is the non-spherical component of the source’s motion (spherically symmetric motions, e.g. a rotating perfect sphere will not radiate as they lack a time-varying quadrupole moment) and M is the mass involved in that motion. It can be seen that even for nearby sources (e.g. $1\;\mathrm{kiloparsec}=3.09\;\times \;10^{19}$ m) with the mass of the Sun (1$M_{⊙}=1.99\;\times \;10^{30}$ kg) moving at near-relativistic velocities ($0.01c=3.00\;\times \;10^{6}$ m/s) the strain at Earth will be $h∼3\;\times \;10^{-20}$, equivalent to a displacement between the Sun and the Earth of ∼5 nm. As of yet no gravitational waves have been directly detected. However, their existence has long been indirectly inferred from the excellent agreement between theory and observation of the orbital decay of neutron-star binary systems (e.g. [2]).

### 1.1 Astrophysical sources of gravitational waves

Any non-axisymmetric acceleration of mass will emit gravitational waves , but as seen above the emission of strong (and potentially observable) gravitational waves requires very large masses and relativistic motion. To fulfil both these requirements, the sources must be compact and dense. The archetypal source is the coalescence of a pair of compact stars (e.g. neutron stars or black holes) in a tight binarysystem. Other sources include: individual rotating neutron stars with sustained distortions on them [3, 4]; vibrating neutron stars [5] and black holes [6]; the collapsing core of a star during a supernova [7] or γ-ray burst [8]; or, more exotically, inflation following the Big Bang [9] and cosmic strings [10, 11]. An excellent review of gravitational wave sources and the science that can be learned from them is presented in [12].

### 1.2 The detection of gravitational waves

The basic principle behind any gravitational wave detector is to measure the relative displacement of freely falling bodies. The first attempts to construct a detector to observe them were made by Joseph Weber in the 1960s [13]. He used resonant mass, or “bar”, detectors consisting of a suspended aluminium cylinder encircled with piezoelectric sensors, and designed to resonate within a narrow frequency band when excited by a passing gravitational wave . Modern bar detectors utilised improved seismic isolation and operated at cryogenic temperatures (≤6 K) to reduce the thermal noise. Detectors including ALLEGRO (Louisiana, US), EXPLORER (CERN), NAUTILUS (Frascati, Italy) and NIOBE (Perth, Australia) have been used to set interesting limits on gravitational wave emission [14]. Following on from these inherently narrow-band “bar” detectors came the broad-band interferometric gravitational wave detectors [15–18], which were pioneered by Rai Weiss at MIT [16]. The aim of these was to use interferometry to measure the displacement of two suspended mirror-coated test masses at the end of each arm. The techniques required to allow these detectors to achieve the extraordinary accuracy necessary to detect a gravitational wave will be detailed within this review. One thing that can be seen straight away from the previous definition of the strain, h, is that larger displacements, or smaller strains, can be measured if the separation of the test masses is large. Therefore, a prerequisite of current and future generations of interferometric gravitational wave detectors is that the arm lengths are long, generally on the few kilometres scale.

### 1.3 Searches for gravitational waves

The most recent efforts to detect gravitational waves have come from the US Laser Interferometric Gravitational-wave Observatory (LIGO) [19, 20], the French-Italian Virgo [21, 22] and the British-German GEO600 [23] projects. LIGO operates two sites (one in Livingston, Louisiana called LLO and one in Hanford, Washington called LHO) each housing an interferometer with 4 km arms and Fabry-Perot cavities (previously LHO also housed a 2 km interferometer). Virgo is a 3 km interferometer with Fabry-Perot cavities in Cascina, Italy. GEO600 (or latterly GEO-HF [24, 25]), located near Hannover, Germany, has 1.2 km arms, but in a folded configuration and with no Fabry–Perot cavities.

Over the last decade, these detectors have produced unprecedented limits on gravitational wave emission from a wide variety of sources. The sensitivity of these detectors has meant that they would be able to observe neutron star binary coalescences out to distances of a few tens of Mpc [26] (a review of expected source rates can be found in [27]). Some highlights of the astrophysics achieved include: surpassing the spin-down limit (assuming the star’s observed slow down is entirely due to loss of energy through gravitational waves ) for the Crab [28, 29] and Vela [30] pulsars; excluding binary coalescences as progenitors of two nearby γ-ray bursts [31, 32]; and, limiting the stochastic gravitational wave background to have an energy density less than $7\;\times \;10^{-6}$ of the closure density of the Universe [33]. A review of many more of the searches can be found in [34].

The LIGO and Virgo detectors are currently undergoing upgrades to their “advanced”, or second generation, configurations: Advanced LIGO (aLIGO) [35] and Advanced Virgo (AdV) [36]. An estimate of the commissioning and observation schedule for these advanced detectors is discussed in [37]. Another second-generation interferometric detector, which will be located underground and utilise cryogenic cooling of its mirror, is the Japanese KAGRA [38] (formerly the Large-Scale Cryogenic Gravitational-wave Telescope, or LCGT [39]). These detectors will aim to increase sensitivity over the initial generation of detectors by a factor of ten, giving a 1000-fold increase the observable volume, in addition to lowering the operating frequency down to ≃10 Hz. Theoretical predictions suggest [27] that the likely number of observable neutron stars coalescences for these detector, once at design sensitivity, could be 40 per year, with the most optimistic estimates suggesting over one per day.

One fundamental limit to the low-frequency (≲1 Hz) sensitivity of ground-based detectors is Newtonian noise, so to observe sources below this (e.g. supermassive black hole binaries [40] and extreme mass ratio black hole inspirals [41]) requires detectors in space like the proposed evolved Laser Interferometer Space Antenna (eLISA) [42, 43], or similar (e.g. [44–46]). Detection of nanoHz gravitational waves from the most massive supermassive black hole systems is also possible via use of high-precision pulsar timing [47], in which pulsars themselves are used as the test masses for a galactic-scale detector. Even lower frequency gravitational waves could be imprinted in the Cosmic Microwave Background (CMB) and be detectable through the polarisation state of the microwave photons. Recent observations by the BICEP2 Collaboration show the first possible evidence for such relic gravitational waves in the CMB [48], although confirmation with other independent observations would strengthen the detection (e.g. with the Planck satellite [49, 50]). We will not discuss any of these techniques further in this review.

## 2 State-of-the-art Interferometer for gravitationalwave detection

**Figure 2.**
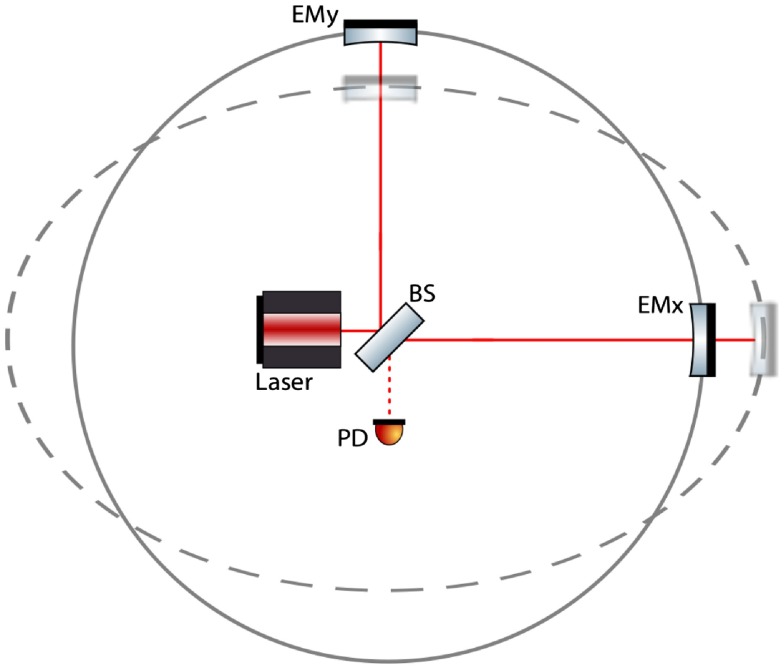
Simplified schematic of Michelson interferometer acting as a gravitational wave detector and the effect of a + polarised wave passing perpendicular through the plane of the interferometer. The light from a laser is split 50:50 by a beam splitter (BS) and enters the two arms of the interferometer. After bouncing back from the end mirrors (EMx and EMy), the light finally recombines on the BS. The interference pattern is detected by a photodiode (PD). In the presence of a gravitational wave, one arm is increased in length while the other is shortened, resulting for a suitably strong gravitational wave in a detectable change of the interference pattern. (The colour version of this figure is included in the online version of the journal.)

In this section, we will give a brief overview of the interferometer layout and configuration of a state-of-the art gravitational wave detector, such as aLIGO [35]. Fundamentally, all these instruments are based on large Michelson interferometers continuously measuring the differential fluctuations in space-time of the two perpendicular interferometer arms, by actually monitoring the light travel time between high-quality mirrors acting as test masses. Due to the fact that gravitational radiation is of quadrupolar nature (see Figure 1), a Michelson interferometer is well suited for observing them. Consider a simple Michelson interferometer as displayed in Figure 2. If, for instance, a gravitational wave of +-polarisation passing perpendicular to the interferometer plane through the Michelson interferometer causes one of the interferometer arms to become shortened, while at the same time the other one will be increased in length. The change in the relative travel time through both arms leads, when the returning beams recombine at the beam splitter, to a change in the interference pattern which can be detected by a photodiode at the output port of interferometer. Carrying out such a differential measurement of the length of the two arms is beneficial, because it allows one to suppress a variety of technical noise sources common in both arms, such as frequency noise of the laser.

State-of-the-art gravitational wave observatories are extremely complex machines, consisting of kilometre long ultra-high vacuum systems, ultra-stable high-power continuous wave lasers, high-performance seismic isolation systems and dozens of super-polished low-loss mirrors suspended in multi-cascaded pendulum systems (see for instance [35, 51]). To establish low-noise operation, these instruments require hundreds of control loops to be closed, making, for example, sure that all mirrors are kept on their longitudinal operation point and are aligned exactly in the right direction.

Though these gravitational wave detectors are technically very complex, their fundamental working principles can be broken down into two general requirements. To build a sensitive gravitational wave detector, first of all one needs to make sure that the test masses are more quiet than the expected effect on the arm length from a passing gravitational wave. This is the reason why we host our instruments inside vacuum systems and take great care to reduce the thermal noise of the mirrors and their suspensions to an equivalent displacement noise below $10^{-20}$m/$\sqrt{\mathrm{Hz}}$ at a frequency of 100 Hz. Secondly, one needs to ensure to be able to read out the mirror positions, i.e. the differential arm length degree of freedom to the required accuracy, while at the same time avoiding introducing any significant level of back-action noise from the measurement process. As we will see below, this point directly relates to the interferometric readout of the test mass position and the associated so-called *quantum noise* (see Section 3.4). In addition to these fundamental sources, there are a variety of technical noise sources, which are kept ≲10% of the fundamental noise sources, including laser frequency and intensity noise, charging noise, scattered light and the facility limits due to residual gas pressure in the vacuum system [20]. The expected limit to performance from the basic noise sources of the aLIGO detector is shown in Figure 6. In order to meet the noise requirement of the advanced detectors, a variety of techniques are used to mitigate the fundamental noise sources as described in Section 3.

### 2.1 Laser sources for gravitational wave detectors

In order to achieve their targeted sensitivity gravitational wave detectors employ ultra-stable, high-power, single-mode, continuous wave laser sources. Good stability is required in terms of low beam jitter noise, high frequency stability and low relative intensity noise. It is worth noting that for some of these noise sources, a good stability is not only required in the frequency range of the gravitational wave detection band, but also at DC as well as at radio frequencies (RF). For instance, the relative intensity noise has to be below a certain level ($<10^{-8}/\sqrt{\mathrm{Hz}}$) in the audioband, because otherwise the power noise would couple either directly to the gravitational wave channel or via radiation pressure acting on the main test masses. At the same time, it is important that the DC power level is very stable over minutes to months time scales to ensure stability of all control loops keeping the mirrors at their operation points. Furthermore, it is also important to have low noise ($<10^{-9}/\sqrt{\mathrm{Hz}}$) in the RF range (about 10 to 100 MHz), as otherwise the noise could couple via the RF modulation sidebands (see Sections 2.3 and 2.4) to the gravitational wave channel. Another requirement for laser sources to be suitable for gravitational wave detectors is the provision of linear, fast and wide-range actuators allowing external frequency and power stabilisation.

While early gravitational wave detector prototype interferometers used Argon ion lasers, nowadays all gravitational wave interferometers employ solid-state lasers emitting at a wavelength of 1064 nm. The preferred transverse electromagnetic mode is the TEM${}_{00}$ mode, i.e. a Gaussian beam. Advanced gravitational wave detectors require laser output powers of the order of 100–200 W.

As a good example of a state-of-the-art laser source, we will in the following briefly describe the Advanced LIGO laser systems [52, 53]. This three-stage system can deliver about 150 W with more than 99.5 % of the power being in the TEM${}_{00}$ mode. The first stage consists of a non-planar ring-oscillator (NPRO) based on a Nd:YAG laser crystal, pumped at a wavelength of 808 nm. The NPRO provides an output power of only 2 W, but due to its monolithic resonator design, it has very low intrinsic frequency noise. The following two stages of the laser system are used to increase the output power, while at the same time inheriting the frequency stability and low noise of the NPRO. The second stage consists of a Nd:YVO${}_{4}$ amplifier with an output power of 35 W. Finally, at the end of the chain a high-power, injection locked, ring-oscillator with four Nd:YAG crystals provides a maximum output power of more than 200 W.

### 2.2 Mode cleaning

In order to provide the required spatial and temporal stability of the laser beam entering the main interferometer, all currently operating gravitational wave detectors make use of mode cleaning cavities [54, 55]. A high-finesse, three-mirror ring cavity is used to filter out geometric noise in the laser beam, i.e. to suppress beam jitter and higher order optical modes.

**Figure 3.**
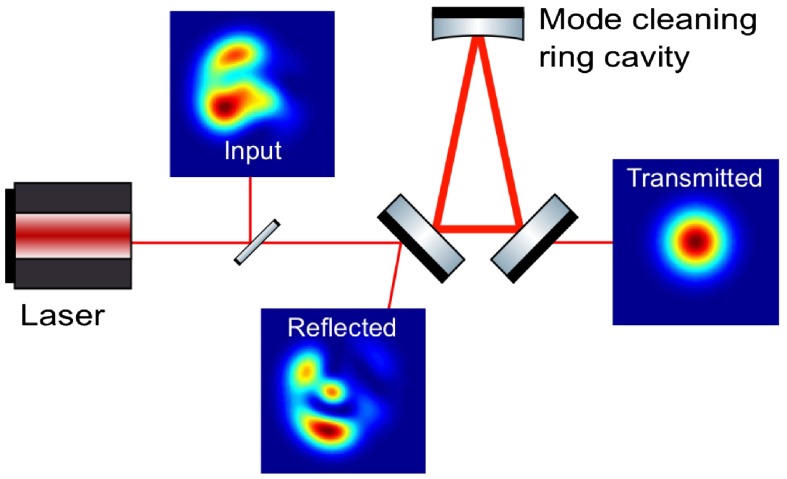
Sketch illustrating the working principle of mode cleaning cavity. The ring resonator is designed to be resonant for the TEM${}_{00}$ mode, but reject higher order TEM mode light. (The colour version of this figure is included in the online version of the journal.)

Figure 3 illustrates the working principle of a mode cleaner cavity. The light incident onto the ring cavity (Input) is not a pure TEM${}_{00}$ beam, but can be strongly distorted, i.e. contain significant amounts of higher order modes, especially first- and second-order modes. The length of the mode cleaner cavity is set so that the fundamental mode of the beam is resonant. Since the Guoy phase shift accumulated in a round trip through the ring cavity depends on the optical mode order, the geometry of the mode cleaning cavity can be designed to be resonant only for the zero-order beam and off resonance for higher order beams. As a result, the input light is split off in two components: While the TEM${}_{00}$ mode is resonating in the mode cleaner and being transmitted towards the main interferometer, the higher order modes are reflected from the cavity. The same effect could also be achieved by a linear two-mirror cavity. However, using a three-mirror cavity allows an easy separation of the input beam and the reflected ‘junk’ light.

For a triangular mode cleaner cavity as displayed in Figure 3, consisting of two flat mirrors positioned close together and a curved mirror at the opposite end of the cavity, the suppression factor $S_{k}$ for a TEM mode of ode order k can be expressed as [56]:

(2) $$\begin{array}{c} S_{k}\approx \sqrt{1+4\frac{F^{2}}{\pi^{2}}sin^{2}\left[k,\arccos,\sqrt{1-\frac{L}{2R}}\right]}, \end{array}$$

where F is the finesse of the ring resonator, L the roundtrip length and R the radius of curvature of the curved mirror. The GEO 600 detector makes use of two consecutive mode cleaner cavities of 8.0 and 8.1 m roundtrip length and finesse values of about 2000, providing an overall suppression factors in the range of $3\times 10^{5}$ to $2\times 10^{6}$ for the first four mode orders [56].

Similar to mode cleaners at the input of the main interferometers, filter cavities are used at the output port of a gravitational wave interferometer, so-called *Output Mode Cleaners (OMC)*, to suppress on one hand higher order modes originating from mirror distortions of the main optics as well as reducing the strength of potential RF modulation sidebands (see the following two sections) [57–59].

### 2.3 Length stabilisation of cavities and interferometers

For the mode cleaner cavities to work as required, their length has to be kept constant to ensure resonance condition for the TEM${}_{00}$ mode. Control loops are used to very accurately position all relevant mirrors at their operating points and to suppress any outside disturbances, such as seismic motion. The error signals for these control loops are usually generated by RF modulation/demodulation techniques derived from the so-called *Pound Drever Hall (PDH)* technique [60].

**Figure 4.**
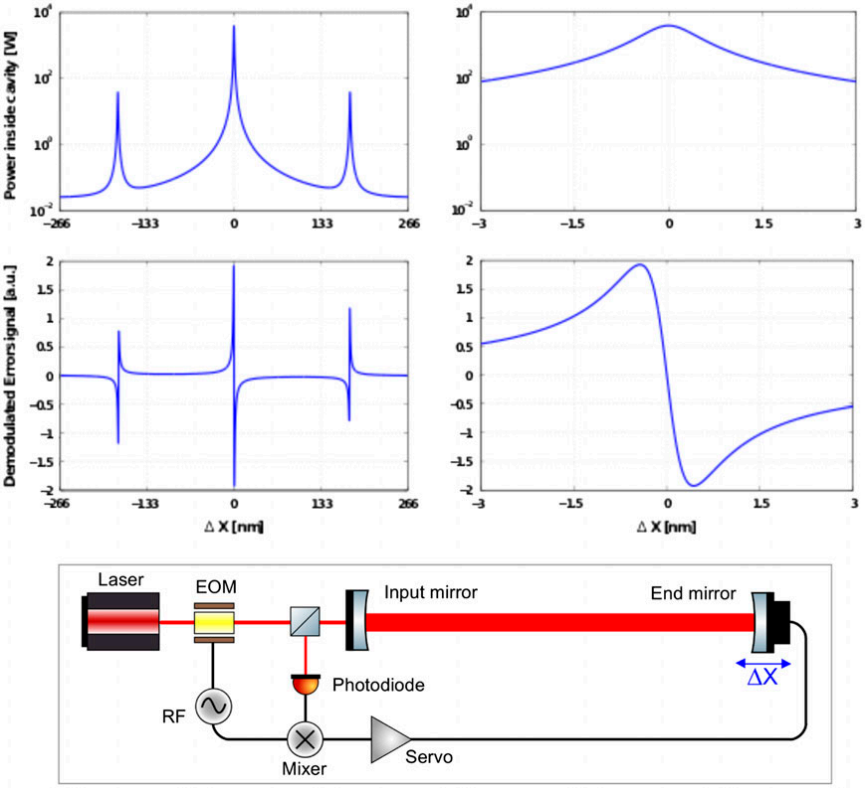
Sketch illustrating the principle of Pound Drever Hall technique used to derive a bipolar error signal for keeping a cavity on resonance for carrier laser light. Refer to the text for a detailed description. (The colour version of this figure is included in the online version of the journal.)

Figure 4 illustrates the principle of the PDH technique. An electro-optic modulator (EOM) is used to imprint RF phase modulation sidebands onto the laser carrier light. The frequency of these sidebands needs to be chosen so that the sidebands are not resonant in the cavity and are directly reflected off the cavity. In contrast, for a cavity length close to resonance, the carrier light will at least partly enter the cavity and encounter a phase shift depending on the microscopic length of the cavity. A photodiode in reflection of the cavity can then be used to detect the beat of the carrier light leaving the cavity and RF sidebands being reflected off the cavity. If the photodiode signal is mixed down with the original RF modulation, a bipolar, linear error signal for the cavity length can be created. The two upper left insets of Figure 4 show the optical power inside the cavity and the demodulated error signal. Three zero-crossings can be obtained, one for each of the sidebands and the carrier being resonant in the cavity. The two upper right insets show the cavity power and the error signal zoomed in around the carrier resonance. The linear range of the error signal, also referred to as locking range, in the shown example is about a fraction of a nanometre.

Modulation/demodulation schemes very similar to the PDH technique can not only be used to control simple cavities, but also for all relevant degrees of freedom of gravitational wave interferometer, such as for instance the differential arm length from which the gravitational wave channel is derived. The required length stability of the differential arm length in advanced gravitational wave detectors is of the order 10${}^{-15}$ m rms and better than 10${}^{-20}$ m$/\sqrt{\mathrm{Hz}}$ at audio frequencies. It is worth noting that the PHD technique can be extended from longitudinal degrees of freedom to alignment degrees of freedom, by using quadrant photodetectors to implement so-called *differential wavefront sensing* [61].

### 2.4 Readout techniques

**Figure 5.**
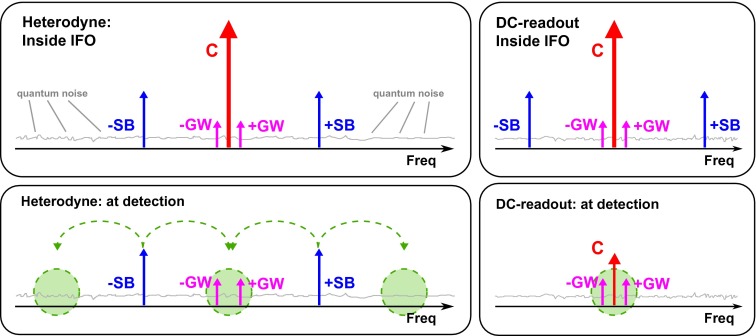
Sketch illustrating the principles of different readout schemes. The two left-hand panels refer to light fields present inside the interferometer (top) and at the main photodetector (bottom) in the case of heterodyne readout, while the two right-hand panels illustrate the case of DC-readout. Abbreviations used are SB = RF sidebands, GW = GW sidebands (audioband) and C = carrier. (The colour version of this figure is included in the online version of the journal.)

We have already mentioned in the previous section that we can readout the differential arm length of a gravitational wave detector by using a RF modulation/demodulation schemes, often also referred to as heterodyne readout. In the following, we want to have a closer look at this technique and briefly discuss some of the relevant noise terms. The upper left box of Figure 5 shows the relevant light fields inside a standard gravitational wave interferometer: the strongest light is the carrier (C), followed by the RF phase modulation sidebands (SB) imprinted in front of the interferometer. If the differential arm length is modulated, for instance due to a gravitational wave signal, then phase modulation signal sidebands (GW) are created in the arms of the interferometer. The lower left box of Figure 5 shows the relevant light fields at the output port of the interferometer: at the output port no carrier light is present, because the interferometer is usually operated at a so-called *dark finge*, i.e. the differential arm length is chosen to give destructive interference for the carrier. However, in contrast to the carrier, the RF sidebands and the gravitational wave sidebands interfere constructively at the beam splitter and leave the interferometer towards the main photodetector. The photodiode signal is then demodulated at the heterodyne frequency to recover an audioband signal containing the gravitational wave signal. This demodulation process is indicated by the green dashed arrows. The three green circles indicate the frequency regions that are beaten down into the gravitational wave channel. Unfortunately, only the green circle in the centre contributes to the signal, while the frequency regions of the outer two green circles just contribute additional quantum noise (see Section 3.4).

Therefore, advanced gravitational wave detectors abandoned heterodyne readout techniques and now employ a so-called *DC-readout* [62–64]. The two right-hand boxes of Figure 5 schematically illustrate the DC-readout principle: inside the interferometer exactly the same light fields are present as in the heterodyne case. However, at the output port of the interferometer, there are two major differences in the DC-readout scheme compared to the heterodyne case: first of all in DC-readout there is a small amount of carrier light present, which is used as local oscillator for the gravitational wave sidebands. The presence of the carrier light is achieved by tuning the differential arm length to be slightly off the dark fringe. The green circle indicates the frequency region that is actually sensed. We see that in the case of the DC-readout, no quantum noise from twice the heterodyne frequency is mixed down into the gravitational wave channel, which significantly improves the signal-to-quantum-noise ratio [65]. So, with DC-readout the heterodyne sidebands do not need to be present at the main photodiode. Therefore, in order to reduce the light power on the photodiode and suppress additional noise couplings, the RF sidebands are filtered out by using an output mode cleaner (see Section 2.2). So, in the case of DC-readout, the output mode cleaner needs to be designed to provide both the filtering of higher order TEM modes as well as suppression of the RF sidebands (which are usually still required for the control of auxiliary degrees of freedom).

## 3 Noise sources in gravitational wave detectors

There are a number of noise sources which limit the sensitivity of long-baseline interferometric detectors in the frequency range 10 Hz–10 kHz. These include fundamental noise sources [51] such as seismic noise, thermal noise in the mirror test masses, suspensions and coatings, and quantum noise.

**Figure 6.**
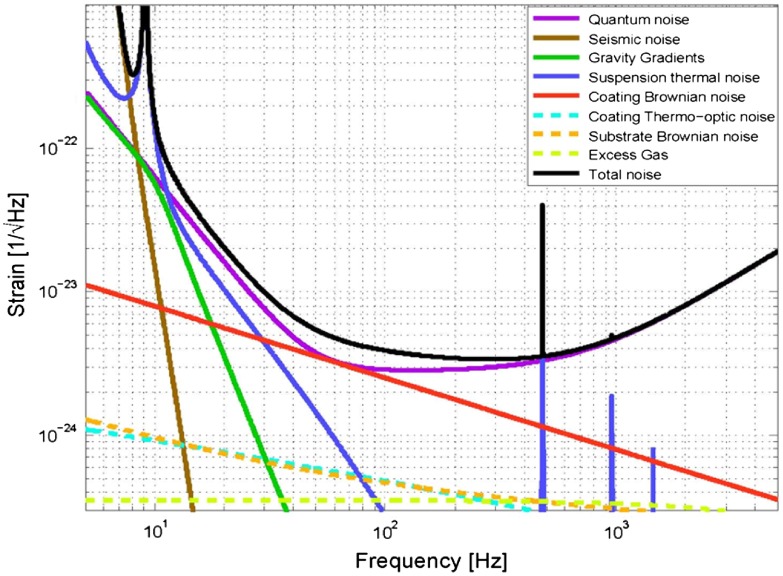
The expected limit to performance from the basic noise sources of the aLIGO detector showing the dominant fundamental noise sources as described in [66]. This figure was produced using the GWINC software [67]. (The colour version of this figure is included in the online version of the journal.)

### 3.1 Newtonian noise and seismic noise

In the low frequency regime (<10 Hz), density perturbations give rise to direct Newtonian couplings to the test masses of the interferometer. This results in a hard limit to the low frequency performance of detectors as the gravitational field from these perturbations cannot be shielded. Fluctuations can be either man-made, such as moving masses, or environmental including density perturbations in the ground due to surface seismic waves. For the advanced detector network, work is currently underway to assess the feasibility of monitoring the local gravitational field gradient at the test mass with an array of accelerometers and/or seismometers and performing subtraction from the data stream either real-time or via post processing [68]. For future detectors, either currently under construction (KAGRA [38]) or proposed (Einstein Telescope [69]), there is the possibility to locate the detectors a few hundred metres underground where the effect due to surface seismic waves is diminished by a factor approximately 10 [69].

**Figure 7.**
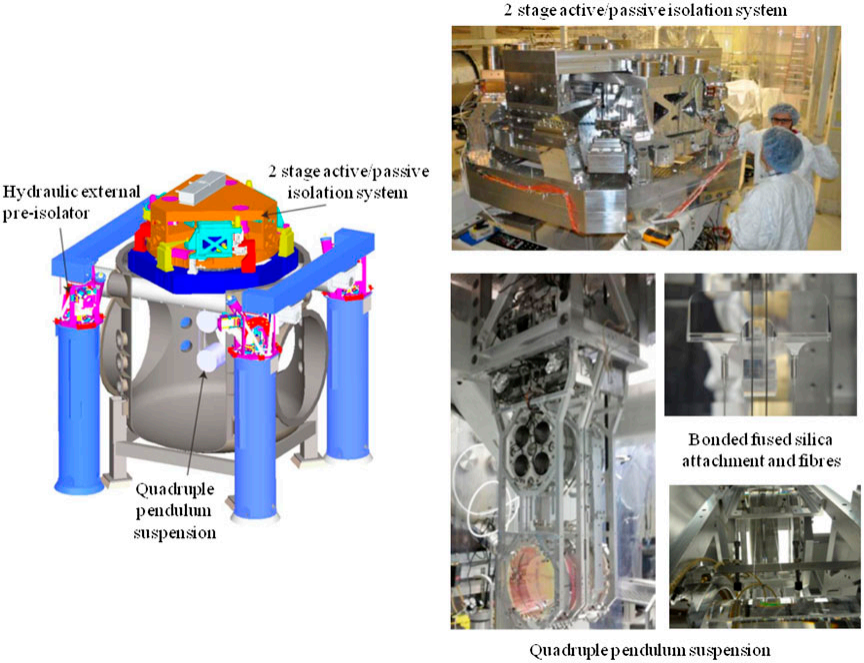
Schematic and photographs of the aLIGO Inertial Seismic Isolation system and quadruple pendulum suspension. (The colour version of this figure is included in the online version of the journal.)

Seismic noise (<10 Hz) is correlated with periods of stormy weather and human activity. For a quiet site, the acceleration spectral density is approximately $10^{-7}/f^{2}\;ms^{-2}/\sqrt{\mathrm{Hz}\}\}}$ [70, 71]. At frequencies around 0.2 Hz, the seismic spectrum is dominated by the microseismic peak, which originates from ocean waves impinging onto the continental plates. Although this is below the operating bandwidth of detectors, it does contribute to the root mean square of the test mass motion and thus must be reduced in order to bring the interferometer mirrors to their operating point. At higher frequencies (>10 Hz), the seismic noise is mainly anthropogenic and the seismic spectrum is highly variable between day and night. Seismic noise is minimised by suspending the test masses of the interferometer from multiple-stage seismic isolation systems. These systems are typically a combination of passive isolation systems and active systems [72, 73] to provide broadband seismic attenuation. It is instructive to consider a mass, m, attached to a spring of stiffness, k with damping constant, b. The transfer function between the ground motion, $x_{g}$ and the motion of the mass, $x_{m}$ is

(3) $$\begin{array}{c} \frac{x_{m}}{x_{g}}=\frac{\omega_{0}^{2}}{\sqrt{{\left(\omega_{0}^{2},-,\omega^{2}\right)}^{2}+\omega^{2}\gamma^{2}}}, \end{array}$$

where $\omega_{0}=\sqrt{k/m}$ is the resonant angular frequency and $\gamma =\left(b,/,m\right)$ is the damping constant. At low frequencies, the ground motion and mass motion are identical and the transfer function becomes unity. In effect, the mass and ground move together. At high frequencies, $\omega \gg \omega_{0}$, the transfer function becomes $\left(\omega_{0}^{2},/,\omega^{2}\right)$ (in the limit of low damping) which provides vibration isolation of the test mass. This is an effective strategy for providing isolation above a few hertz. In AdV [72], the seismic isolation system comprises multiple (≃7) stacks of low frequency (≃0.1Hz) vertical isolators, and a combination of an inverted pendulum and pendulum stages for horizontal isolation. For aLIGO [73], the platform to be isolated is suspended with stiff springs (≃5Hz) and implemented with seismometers which monitor the platform motion in all six degrees of freedom. The output of the seismometers is driven to zero via feedback control to voice-coil actuators, thus providing isolation from the unity gain bandwidth of approximately 0.1 Hz up to the point where passive isolation becomes effective (a few hertz). Figure 7 shows the two stage active/passive seismic isolation platform utilised in aLIGO which provides a factor of 10 isolation at the microseismic peak and a factor of 1000 isolation in the 1-10 Hz range [74]. The aLIGO detector further includes an out of vacuum hydraulic actuator which permits large low frequency motion at the level ±1 mm. This is an effective technique to take out large low frequency changes in the length of the interferometer arms due for example to earth tides (≃600μm variation at a frequency of ≃23.1μHz).

**Figure 8.**
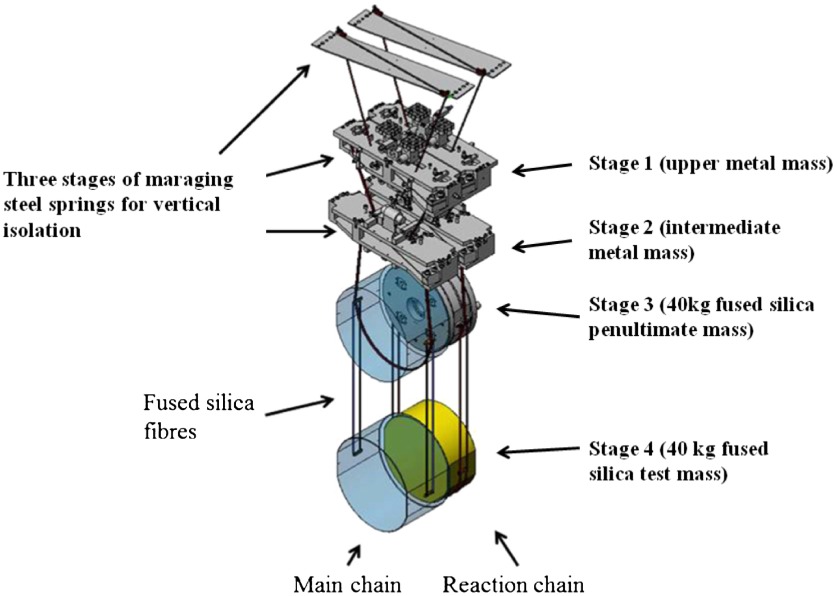
Schematic of the aLIGO quadruple pendulum suspension. The 40 kg test mass is the lowest mass in the main chain, while a parallel reaction chain allows for control forces to be applied from a quiet reference frame. (The colour version of this figure is included in the online version of the journal.)

### 3.2 Suspension thermal noise

The test masses of interferometric gravitational wave detectors are supported by multiple-stage suspensions that hang below the seismic isolation platforms (see Figures 7 and 8). The role of the suspension is to further isolate the test mass from seismic noise in addition to minimising thermal noise. For aLIGO, a quadruple pendulum system [75] is utilised which comprises four pendulum stages for horizontal isolation and three stages of cantilever springs to provide vertical isolation. The pendulum further allows the application of control forces for the purpose of alignment and control. These forces are often termed *local* when they provide damping of a given pendulum suspension or *global* where the forces are used to control the length and angular degrees of freedom of the interferometer optical cavities [75]. In order not to spoil the seismic attenuation, the forces are applied from a parallel chain called the reaction chain. The control forces are applied in a hierarchical way [76] such that large forces are applied to the upper stages of the suspension via coil-magnet actuator, and smaller forces (with peak forces in the μN range) are applied directly to the test mass via an electrostatic actuator.

To minimise the thermal noise associated with the pendulum modes of the suspension, the final stage consists of a silica mirror, 40 kg in mass (the test mass), suspended from another silica mass (the penultimate mass) by four silicafibres. These fibres are fabricated using a CO${}_{2}$ laser as described in [77, 78]. Suspension thermal noise arises due to mechanical dissipation in the materials which make up the suspension (Brownian noise) or via coupling of statistical temperature fluctuations through the thermo-mechanical properties of the suspension materials (Thermoelastic noise) and is conveniently calculated via the Fluctuation-Dissipation theorem [79]. Above the pendulum resonance, the displacement thermal noise ($x_{\mathrm{susp}}$) due to the suspension is given by

(4) $$\begin{array}{c} x_{\mathrm{susp}}^{2}=\frac{4k_{B}T\omega_{0}^{2}\phi_{\mathrm{total}}}{m\omega^{5}}, \end{array}$$

where T is the temperature, m is the pendulum mass, $\phi_{\mathrm{total}}$ is the mechanical loss of the pendulum (∝1/Q with Quality factor Q), $\omega_{0}$ is the resonant angular frequency, $k_{B}$ is Boltzmann’s constant and ω is the angular frequency. Application of Equation (4) should be applied for all resonant modes of the suspension, although in principle the pendulum mode (≃0.6 Hz) and the vertical bounce mode (≃9 Hz), which enters via a 0.1% cross-coupling, are the dominant terms.

To minimise the displacement thermal noise in room temperature detector requires the use of ultra-low dissipation materials with low loss angle such as fused silica. The final stage consists of the fused silica test mass attached to the fused silica penultimate mass via four fused silica fibres approximately 400μm in diameter and 60 cm long [78]. The fibres are welded to silica ears which are attached to the sides of the mass using hydroxide-catalysis bonding (silicate bonding) [80, 81]. The final stage is thus a monolithic fused silica suspension exhibiting extremely low thermal noise. The dominant contributions to the mechanical loss, and thus thermal noise performance, will now be briefly discussed. Surface loss [82, 83] originates from defects on the surface such as dislocations, un-terminated dangling bonds and surface cracks and is an important loss mechanism for fibres which have a high surface to volume ratio

(5) $$\begin{array}{c} \phi_{\mathrm{surface}}=\frac{4h\phi_{s}}{r}, \end{array}$$

where $h\phi_{s}$ is related to the level of surface damage on the fibre surface and r is the fibre radius. Thermoelastic loss [84] arises from the fact that bending a suspension fibre leads to heating/cooling via the thermal expansion coefficient. Heat flow across the fibre leads to dissipation. When the fibre is under tension, the variation in Young’s modulus with temperature leads to an additional thermoelastic contribution. For fused silica, these two terms have opposite sign and the thermoelastic loss can be cancelled in the bending region by suitable choice of the fibre geometry [85]. Thermoelastic loss is given by

(6) $$\begin{array}{c} \phi_{\mathrm{thermoelastic}}=\frac{YT}{\rho C}{\left(\alpha ,-,\frac{\sigma \beta }{Y}\right)}^{2}\left(\frac{\omega \tau }{1+{\left(\omega ,\tau \right)}^{2}}\right), \end{array}$$

where Y is the Young’s modulus of the fibre, C is the specific heat capacity of the material per unit mass, ρ is the density, α is the coefficient of thermal expansion, σ is the static stress in the fibre due to the suspended load, $\beta =\left(1,/,Y\right)\left(d,Y,/,d,T\right)$ is the thermal elastic coefficient and T is the temperature. The characteristic time for the heat flow across the fibre is defined as τ which for a circular cross-section fibre is [84]

(7) $$\begin{array}{c} \tau =\frac{1}{2.16\pi }\frac{\rho Cr}{\kappa }, \end{array}$$

with thermal conductivity κ. For the advanced detectors (aLIGO and AdV), the fibre design is carefully chosen such that at the bending point of the suspension, the tensile stress is chosen to null the thermoelastic noise contribution for the pendulum mode. The remaining dominant mechanical loss terms are weld loss and bond loss. Weld loss arises from material which has been heated with a $CO_{2}$ laser [78] to fuse the silica suspension fibres to the attachment ears on the side of the test mass. This material exhibits a loss which is higher than the bulk loss [86] and likely correlated with the level of thermal stress. Bond loss arises from the fused silica ears silicate bonded to the side of the test mass. The silicate bonding process produces a strongly cross-linked structure which allows glassy materials to be reproducibly attached in a mechanically and thermally stable way [87].

A pendulum mirror suspension stores energy both in the elasticity of the fibre material and the gravitational field. The latter term is lossless and dominates in heavily loaded suspension fibres. This implies that the pendulum loss is lower than that of the material used for the suspension fibre. This property is termed dissipation dilution D and allows the total mechanical loss to be diluted to $\phi_{\mathrm{total}}=\phi /D$. The mechanical loss, ϕ, is conveniently calculated via Finite Element methods which evaluate the loss contributions at each point of the suspension (surface loss, thermoelastic loss, weld loss, bond loss) and scale the loss with the appropriate bending energy stored at that point in the suspension (e.g. a lossy region with zero stored bending energy will not contribute to the loss) [88]. The total diluted loss, $\phi_{\mathrm{total}}$, is then determined by summing all contributions over the entire weld/fibre and used to calculate the thermal displacement noise in Equation (4) for all modes of the suspension stage.

### 3.3 Mirror and coating thermal noise

The mirrors used in the advanced detectors are 40 kg to ensure that radiation pressure noise at low frequencies is sufficiently lowered. The mirrors are cylinders of high-quality fused silica with a surfaces roughness of ≃0.16 nm rms and a radius of curvature of roughly 2 km [35]. The aspect ratio of the mirrors is chosen such that the internal resonant modes are sufficiently high frequency (≃10 kHz and above) to minimise thermal noise and that flexing of the mirror due to the applied Gaussian laser beam does not introduce significant coating thermal noise. The resonant modes of the mirror substrate have extremely high quality factors in excess of $10^{7}$ [83]. These high frequency modes store the majority of the $1/2k_{B}T$ of thermal energy and the off-resonance thermal noise, in the detector band 10 Hz–10 kHz, is sufficiently lowered. For advanced detectors, the mirror substrate thermal noise is significantly lower than the contribution due to the coating thermal noise.

#### 3.3.1 Mirror thermal noise

Mirror thermal noise originates from mechanical loss in the fused silica substrates and comprises contributions from surface loss, thermoelastic loss and bulk loss. The origins of surface and thermoelastic loss have been described in the previous section, while bulk loss originates from the fact that the strained Si-O-Si bonds in fused silica have two stable minima at different bond angles. Redistribution of the bond angles under thermal fluctuations leads to mechanical dissipation. The technique proposed by [89] is a convenient method to estimate both mirror substrate and coating thermal noise. A force, $F_{0}$, applied onto the front surface of the test mass, in this case a Gaussian pressure originating from the laser beam, produces a strain energy ϵ and a dissipated power $W_{\mathrm{diss}}=2\pi f\int_{V}\epsilon \phi dV$ due to mechanical loss ϕ. The power spectral density of the thermal displacement noise is then determined from

(8) $$\begin{array}{c} x_{\mathrm{thermal}}^{2}=\frac{2k_{B}TW_{\mathrm{diss}}}{\pi^{2}f^{2}F_{0}}. \end{array}$$

This technique is convenient as it can handle non-homogeneous systems whereby the loss can be varied as a function of position. This case is particularly useful for modelling both surface loss and bulk loss of the mirror substrate whereby the appropriate strain energy in different parts of the mirror can be determined. It is however instructive to consider closed form analytical solutions and for the case of a semi-infinite mirror substrate, the thermal noise due to bulk dissipation (or Brownian dissipation) may be written [90]

(9) $$\begin{array}{c} x_{\mathrm{mirror}}^{2}=\frac{2k_{B}T}{\sqrt{\pi^{3}}f}\frac{\left(1,-,\sigma^{2}\right)}{Yw_{m}}\phi_{\mathrm{substrate}}, \end{array}$$

where σ is the Poisson’s ratio and $w_{m}$ is the radius of the beam where the electric field has fallen to 1/e. Utilising large radius laser beams is clearly an advantage in order to average over a larger surface area. The mechanical loss of the substrate $\phi_{\mathrm{substrate}}$ as determined from a semi-empirical model has been used to describe the loss contribution in fused silica mirror substrates [83]

(10) $$\begin{array}{c} \phi_{\mathrm{substrate}}=C_{1}\left(\frac{S}{V}\right)+C_{2}{\left(\frac{f}{1\;\mathrm{Hz}}\right)}^{C_{3}}, \end{array}$$

where V is the sample volume, S is the surface area and f is the frequency. The coefficients $C_{1}$ and $C_{2}$ and exponent $C_{3}$ relate to the specific type of fused silica and typical values are $C_{1}=\left(6.5\;\pm \;0.2\right)\times 10^{-9}$, $C_{2}=\left(6.3\;\pm \;0.2\right)\times 10^{-12}$, $C_{3}=0.77\;\pm \;0.02$ [83]. An analytical form for the thermoelastic noise, which couples via the thermal expansion coefficient α, may also be determined

(11) $$\begin{array}{c} x_{\mathrm{thermoelastic}}^{2}=\frac{4\left(1,+,\sigma^{2}\right)\kappa \alpha^{2}k_{B}T}{\pi^{2.5}C^{2}\rho^{2}w_{m}^{3}f^{2}}. \end{array}$$

Corrections which take into account finite-sized mirrors [91] are also readily calculated although for the advanced detector mirror substrates corrections, $C_{\mathrm{fsm}}$, are ≃0.98. Again utilising a large radius laser beams is clearly advantageous to average over a larger surface area of the mirror. For the advanced detectors, the contribution due to the mirror thermoeasltic noise is negligible and thus not shown in Figure 6.

#### 3.3.2 Coating thermal noise

Coating thermal noise originates from the multilayer dielectric coating stacks which make up the high reflectivity coatings on the test masses. The noise contribution can be reduced by utilising low mechanical loss coatings and by averaging over a larger surface of the mirror by utilising larger laser beam diameters. For advanced detectors, a beam radius (1/e) of the order ≃5-6 cm is used on the mirrors as a compromise between lowering coating thermal noise and reducing clipping losses to below 1 ppm and cavity instabilities [45]. The coating layers are made up of alternating stacks of silicon dioxide (SiO${}_{2}$), or silica, as the low refractive index layer and tantalum pentoxide (Ta${}_{2}$O${}_{5}$), or tantala, as the high refractive index layer. The tantala/silica coating material exhibits mechanical losses $3\times 10^{-4}$/$5\times 10^{-5}$, respectively, [90, 92] which is somewhat higher than the fused silica used in the mirror substrates and suspensions. This results in coating thermal noise being an important contribution in the frequency around 75-100 Hz. Significant progress has been made in lowering the thermal noise in coating layers via the additional of dopants including titanium dioxide (TiO${}_{2}$), or titania, in the tanatala coatings [93]. These dopants at the level of ≃25% have been shown to lower the mechanical loss by approximately 40%. There has also been significant progress in understanding the link between mechanical loss and coating microstructure [94] and this is an evolving area in which the fields of gravitational wave detection and solid-state physics are efficiently collaborating to provide low-loss high-reflectivity mirror coatings. This work also has significant applications in the field of precision metrology where low thermal noise mirror coatings are necessary. Expressions for the Brownian and thermoelastic coating thermal noise can be derived for multilayer dielectric coatings to yield for semi-infinite substrates [90]

(12) $$\begin{array}{c} x_{\mathrm{coating}}^{2}=\frac{2k_{B}T\phi_{\mathrm{eff}}\left(1,-,\sigma \right)}{\pi^{3/2}fw_{m}Y}, \end{array}$$

with

(13) $$\begin{array}{c} \phi_{\mathrm{eff}}=\phi +\frac{d}{w_{m}\sqrt{\pi }}\left(\frac{Y}{Y_{⊥}},\phi_{⊥},+,\frac{Y_{\|}}{Y\phi_{\|}}\right), \end{array}$$

where ϕ and Y denote the mechanical loss and Young’s Modulus of the substrate, and the subscripts ⊥ and ‖ denote the perpendicular and parallel components of the coating parameters respectively.

Thermo-optic noise in the coating is treated as contribution from thermoelastic noise due to deformation of the coating surface and thermorefractive noise arising from variations in the refractive index of the coating with temperature fluctuations [95]. This effect is analogous to the suspension thermoleastic noise which can be nulled by suitable choice of the fibre dimension as described in Section 3.2. For advanced detector coatings, the thermorefractive noise can be written

(14) $$\begin{array}{c} x_{\mathrm{thermoelastic}}^{2}=\frac{2\sqrt{2}k_{B}T^{2}}{\pi^{3/2}w_{m}^{2}\sqrt{\kappa Cf}}\left(C_{\mathrm{fsm}},\alpha ,d,-,\beta ,\lambda \right), \end{array}$$

where symbol definitions are identical to Section 3.2, $C_{\mathrm{fsm}}$ is the finite mirror correction factor (≃0.98), d is the coating thickness, β=dn/dT is the variation in refractive index with temperature and λ is the wavelength.

The advanced detectors will utilise optimised coating thicknesses that are slightly different to the standard λ/4 dielectric stacks [96]. This results from the fact that it is the combination of mechanical loss multiplied by coating thickness that determines the level of thermal noise contribution (Equation (13)). As the losses of the tantala and silica coatings are different, an optimum can be achieved that is slightly different to a standard quarter wave layer thickness. The reader is referred to Figure 6 where the dominant noise terms are plotted as a function of frequency.

### 3.4 Quantum noise

Quantum noise plays a very special role in gravitational wave interferometers. First of all, as can be seen in Figure 6, quantum noise is the limiting fundamental noise source over most frequencies in the aLIGO detection band, and therefore it is of ultimate importance to further improve it. Secondly, as indicated by its name quantum noise directly arises from the quantum nature of photons. Two different noise mechanisms contribute to quantum noise:

- *Photon shot noise*   Due to the fact that the photons in a laser beam are not equally distributed in time, but follow a Poisson distribution, any laser beam detected by a photo diode will cause the output of the photo diode to carry noise. This shot noise, which scales proportional to the square root of the detected optical power, can be considered as the readout or sensing noise of the interferometer.
- *Quantum radiation pressure noise*   When the laser beam is reflected by a mirror, the photons transfer momentum onto the mirror, or in other words there is radiation pressure force acting on the mirror. Since, as stated above, the photons are not equally distributed in time, the radiation pressure force on the mirror fluctuates. However, due to the fact that the mirrors in gravitational wave detectors are suspended from a chain of pendulums, the mirrors are susceptible to the radiation pressure force fluctuations and as a consequence the mirror position fluctuates. This quantum radiation pressure noise can be considered as a form of back-action noise from the measurement process itself.

It is interesting to compare how these two quantum noise components scale for different optical power. We have mentioned that the shot noise detected by a photo diode increases with the square root of the optical power. However, because the actual gravitational wave signal scales linearly with the optical power, overall we can win in the gravitational wave signal to shot noise ratio by increasing the power, P. The amplitude spectral density of shot noise equivalent strain follows:

(15) $$\begin{array}{c} h_{\mathrm{sn}}\propto \frac{1}{\sqrt{P}}\;. \end{array}$$

In contrast, the contribution of quantum radiation pressure noise increases linearly with the optical power. The amplitude spectral density of radiation pressure noise equivalent strain is given by:

(16) $$\begin{array}{c} h_{\mathrm{sn}}\propto \frac{P}{mf^{2}}\;, \end{array}$$

where m is the mass of the mirror and f is the frequency.

The opposite scaling of the two quantum noise components with respect to the optical power circulating in the optical system, gives rise to the so-called standard quantum limit (SQL) [97], which can be interpreted to be the equivalent of the Heisenberg Uncertainty Principle applied to interferometry of uncoupled test masses. The higher the optical power, the lower the shot noise contribution and therefore the better our readout accuracy, while at the same time we increase the back action noise and therefore disturb the mirror positions. The SQL poses a fundamental sensitivity limit for any classical interferometer. However, as we will discuss below, there are various non-classical techniques available which potentially allow one to surpass the standard quantum limit.

**Figure 9.**
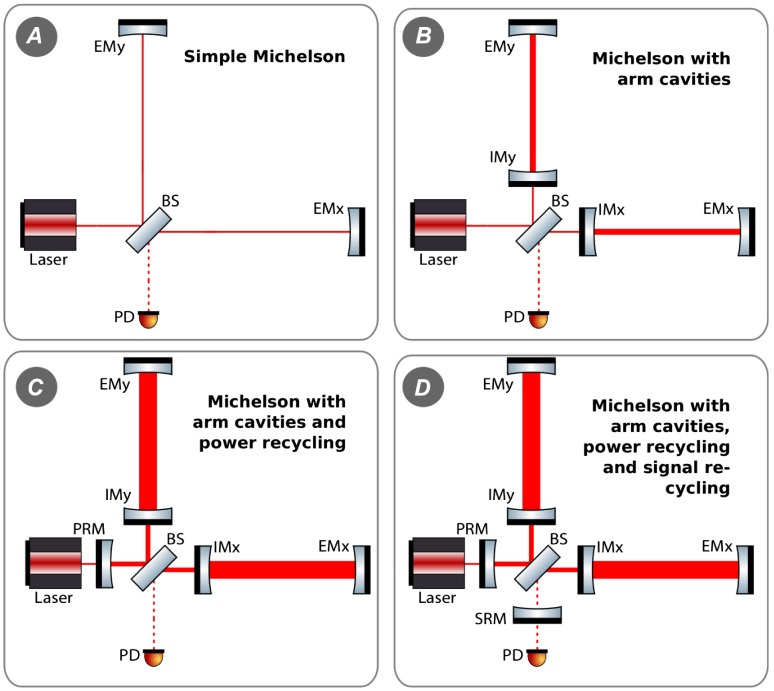
Evolution of interferometer layouts from a simple Michelson (*A*) to advanced gravitational wave detectors as advanced LIGO (*D*). The following abbreviations have been used: BS = beam splitter, IM = input mirror, EM = end mirror, PD = photo detector, PRM = power recycling mirror and SRM = signal recycling mirror. (The colour version of this figure is included in the online version of the journal.)

For the moment let us go back to aLIGO and discuss its interferometric configuration, as shown in the lower right panel of Figure 9. Clearly the aLIGO optical layout is more complicated than the simple Michelson interferometer shown Figure 2, and this is what we should understand.

**Figure 10.**
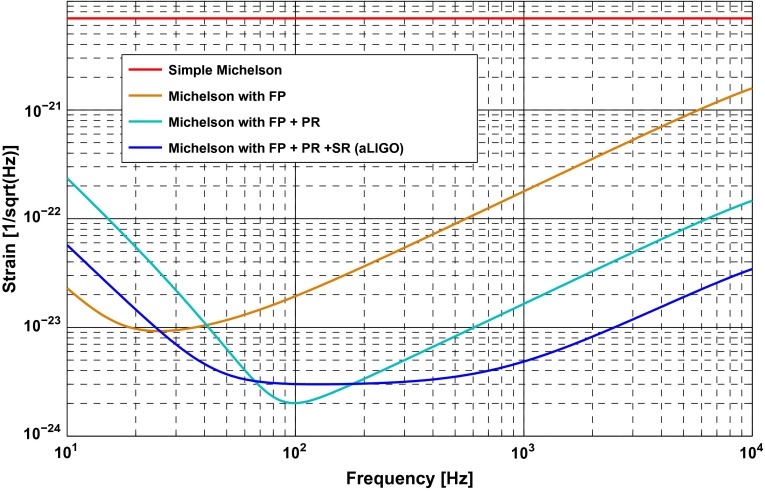
Quantum noise limited strain sensitivities for the four interferometer configurations displayed in Figure 9. (The colour version of this figure is included in the online version of the journal.)

Let us start from a simple Michelson interferometer with 4 km long arms (see panel *A* of Figure 9) and a laser that the delivers 125 W of input power [53]. It is most practical to stabilise (so-called *locking*) the differential arm length of the interferometer so that the beams returning from the two arms interfere completely destructive at the beam splitter. Therefore, in normal operation and in absence of any strong gravitational waves there will be no light at the output port and on the photo diode. Only when there is a change in the differential arm length of the Michelson (maybe due to the presence of a sufficiently strong gravitational wave, or due to an external disturbance acting on the mirrors), the destructive interference will be partly destroyed and light emerges at the output of the interferometer. The corresponding quantum noise limited strain sensitivity of such a simple Michelson interferometer is shown as the red trace in Figure 10; such an instrument would be limited entirely by shot noise and would provide a flat spectrum at a level of $6\times 10^{-21}/\sqrt{\mathrm{Hz}}$.

In order to increase the interaction time of the laser light with a potential gravitational wave most advanced gravitational wave detectors employ Fabry-Perot cavities in the arms to resonantly enhance the light power in the arms (see panel *B* of Figure 9). In the case of aLIGO, these arm cavities feature a finesse of about 400. The resulting sensitivity improvement can be seen in the orange trace in Figure 10. At the sweet spot of 20 Hz the quantum noise limited sensitivity improves by about a factor of 600 compared to a simple Michelson interferometer. It is also obvious that the sensitivity obtained with arm cavities is not flat anymore; above 30 Hz the sensitivity rolls off due to the presence of the arm cavity pole. In addition, using the arm cavities we have increased the laser power so strongly that below 25 Hz quantum radiation pressure noise starts to dominate quantum noise.

In a next step to further enhance the light power inside the interferometer, we apply a technique called power recycling [98] (see panel *C* of Figure 9). As we have discussed above, when set to destructive interference no light is leaving the interferometer towards the output port. For energy conservation reasons this means that all the light returning from the interferometer arms interferes constructively into the input port, i.e. the light goes back towards the laser. This light which would be wasted otherwise can be recycled by placing a semi-transparent mirror, the power recycling mirror (PRM), between the laser and the beam splitter. The effect of power recycling on the quantum noise limited strain sensitivity is displayed in the cyan curve of Figure 10. Due to increasing the circulating light power in the interferometer arms to 0.8 MW we can significantly improve the sensitivity above 100 Hz. However, since we have increased the contribution of quantum radiation pressure noise at low frequencies by the same amount we have reduced shot noise at high frequency, we overall lose sensitivity below 40 Hz.

Finally, all advanced gravitational wave detectors will also make use of a technology called signal recycling [99]. Similarly, to how power recycling recycles the main carrier light leaving towards the input port, by inserting a signal recycling mirror between the beam splitter and the output port (see panel *D* of Figure 9), we can recycle the signal sidebands leaving the interferometer towards the output port. aLIGO employs a specific flavour of signal recycling called resonant sideband extraction. The aim here is to widen the arm cavity bandwidth for the signal sidebands and therefore to more easily extract the side bands by forming a coupled three mirror cavity of SRM, IM and EM, to provide a reduced effective cavity finesse for the signal sidebands. The effect of this technique applied to the aLIGO configuration can be seen in the blue trace of Figure 10. Using signal recycling allows us to significantly widen the instrument response by improving the sensitivity on the low and at the same time high frequency end of the detection band. The application of signal recycling is planned for all future gravitational wave detectors currently in construction or under consideration.

In order to further improve the quantum noise limited strain sensitivity of future gravitational wave detectors, and eventually surpass the SQL, three different concepts have been suggested:

• The injection of squeezed vacuum states
• Techniques exploiting opto-mechanical rigidity
• Speedmeter configurations

#### 3.4.1 Squeezed vacuum

The presence of quantum noise in a laser-interferometric gravitational wave detector can also be understood by vacuum fluctuations of the electrical field entering the interferometer through any open port. For instance the output port of the interferometer can be considered as an open port which allows the vacuum fluctuations to enter the gravitational wave detector, interact with its mirrors (back-action noise) and finally be detected on the main photo detector (sensing noise).

**Figure 11.**
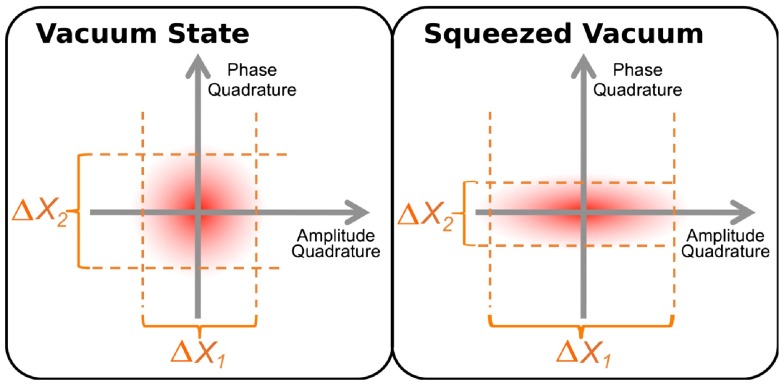
Quantum noise can also be described as vacuum fluctuations. The left panel shows a classical vacuum state with a circular probability distribution. A squeezed vacuum state (right panel) can be used to reduce the vacuum noise one readout quadrature, while increasing the noise present in the other quadrature. (The colour version of this figure is included in the online version of the journal.)

As displayed in the left-hand panel of Figure 11, the vacuum fluctuations feature a circular shape in the quadrature picture. It has to be noted that for a pure vacuum state the noise in the amplitude and phase quadratures is uncorrelated. The radiation pressure noise measured in the gravitational wave detector scales with the noise in the amplitude quadrature, while the shot noise component of the quantum noise scales with the noise in phase quadrature. Therefore, it is obvious that we can redistribute or shape the spectrum of the quantum noise limited sensitivity by changing the shape of the vacuum fluctuations.

While the Heisenberg Uncertainty Principle forbids us to reduce the overall area of this circle, the application of squeezing [100] allows us to manipulate the shape of the vacuum noise and to transform the circle into a squeezed ellipse (see right panel of Figure 11). Experimentally, such states can be realised using non-linear optical technologies. In a first step, some 1064 nm light is taken off the main laser and is converted into frequency doubled light with a wavelength of 532 nm using a second harmonic generation (SHG). This light is then fed into an optical parametric oscillator (OPO), where parametric down conversion is used to created a correlated pair of 1064 nm photons from a single 532 nm photon. If the OPO is operated below threshold, squeezed vacuum states are created [101].

The application of squeezed light to enhance the sensitivity of gravitational wave observatories has been matured over the past decade. The GEO 600 interferometer makes regular use of the injection of squeezed light states since the year 2010 [102] and was used to demonstrate long-term application of squeezing as well as for long duration noise studies [103]. Recently also in one of the LIGO interferometers squeezing tests have been performed to demonstrate a sensitivity improvement at the mid to low-frequency end of the detection band [104].

**Figure 12.**
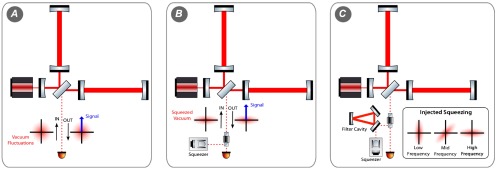
Illustrations of a gravitational wave detector without any squeezing (*A*), the injection of phase-squeezed vacuum states (*B*) and the injection of squeezed vacuum states with a frequency dependent squeezing angle (*C*), which can be obtained by using the dispersion of a filter cavity from which the squeezed light is reflected. (The colour version of this figure is included in the online version of the journal.)

Figure 12 schematically illustrates the injection of squeezed states into a gravitational wave detector. Phase-squeezed vacuum is created by the squeezing source and injected via a Faraday isolator at the output port of the interferometer. The squeezed vacuum state is reflected from the interferometer and, after passing again through the Faraday isolator, is detected together with any potential gravitational wave signal on the main photodetector. A comparison of the output fields in the left and centre panels of Figure 12 show an increased signal (blue arrow) to noise (red ball/ellipse) ratio for the injection of squeezed light.

#### 3.4.2 Opto-mechanical rigidity

The high light powers employed by gravitational wave detectors create significant radiation pressure effects. Forces acting on the test masses originating from light pressure can be of the same order or even larger than mechanical restoring forces acting on the test mass. Therefore, such interferometers are described as *opto-mechanical* systems rather than just mechanical or optical systems. For instance, in a cavity slightly off resonance the circulating light power strongly depends on the relative position of the cavity mirrors. For small changes, the cavity power and therefore also the force acting on the mirrors is linearly proportional to the relative position of the mirrors. The resulting opto-mechanical behaviour resembles spring characteristics similar to Hooke’s law (but with negative damping).

Such optical springs [105, 106] can cause various types of challenges and instability problems for lock-acquisition and controlling an advanced gravitational wave detector (for instance Sidle-Sigg instability [107]). However, on the other hand optical springs can also be utilised to further improve the quantum noise limited sensitivity of interferometers and even to surpass the SQL.

**Figure 13.**
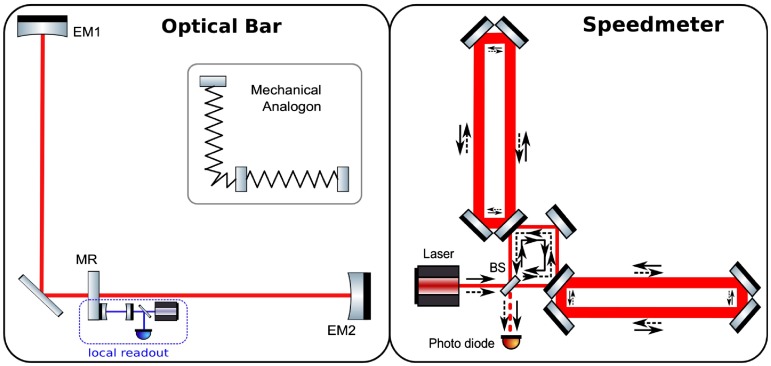
Schematic layouts of an Optical Bar configurations (left) and a Sagnac interferometer-based speedmeter configuration (right). (The colour version of this figure is included in the online version of the journal.)

The left panel of Figure 13 shows the simplest example of an optical spring enhanced gravitational wave detector, a so-called *optical bar* [108]. The central mirror MR is connected to the two end mirrors (EM1 and EM2) via optical springs. In case of a gravitational wave of plus polarisation incident perpendicular to plane of the detector, the distance between MR and one of the end mirrors will be stretched so that the spring in this arm will pull MR, while the distance between MR and the other end mirror will be shortened so that the spring in this arm will push MR. Hence, the optical bar acts as an transducer, which ’converts’ the gravitational wave into a actual movement of MR in respect to its local frame. The gravitational wave signal can then be read out by an independent local readout. The optical bar scheme actually provides two significant benefits. First of all, the test masses are converted from free-falling objects into oscillators, providing increased response for signals at the optical spring resonance. Secondly, separating the gravitational wave transducer from the readout provides us with the possibility to individually optimise the light powers in each of the systems and therefore, if well designed, providing sensitivities below the SQL.

More complex configurations employing optical springs include optical lever [109, 110] and multiple-optical spring configurations (see for instance [111–113]).

#### 3.4.3 Speedmeter topologies

The SQL arises during the measurement of our test masses because the test mass positions at different times ($x(t_{1})$ and $x(t_{2})$) do not commute, i.e.

(17) $$\begin{array}{c} {}[x\left(t_{1}\right),x\left(t_{2}\right)]\neq 0 \end{array}$$

This limitation is imposed by quantum mechanics as soon as we measure the absolute position of mirrors (at DC frequency). However, for gravitational wave detection on the Earth we are anyway only interested at frequencies above about 1 Hz, so that in principle we do not necessarily need to measure absolute positions of mirrors to very high precision.

For example, if we were to measure the speed (or better the momentum, p) of an ensemble of test masses and made sure to not read out any absolute position information, we could in principle beat the SQL over a very wide frequency range. In simple cases, momentum can be considered as a conserved observable and hence we can measure the momentum of an ensemble of test masses continuously with much higher precision than the test mass positions:

(18) $$\begin{array}{c} {}[p\left(t_{1}\right),p\left(t_{2}\right)]=0 \end{array}$$

Knowing the speed of the test masses still allows to reconstruct the gravitational wave signal, while such a measurement would in first order not be limited by the SQL. This concept, coined *speedmeter*, had first been proposed in the context of readout of traditional mechanical bar detectors [114], before it was realised that a Michelson interferometer can be transformed to a speedmeter by adding a so-called *sloshing cavity* at its output [115]. In 2003, a theoretical analysis by Chen [116] identified the intrinsic speedmeter properties of a Sagnac interferometer.

The right panel of Figure 13 shows the optical layout of a Sagnac speedmeter suitable for gravitational wave detection. The key difference between a Michelson interferometer and Sagnac interferometer is that, while in the Michelson all photons only enter one of the two interferometer arms, in the Sagnac speedmeter the photons travel through both arms. Therefore, each mirror is sensed at two different times in quick succession, once by the beam transmitted through the beam splitter (BS) and once by the beam reflected from BS. This is the reason why only the velocity or momentum information of the test masses is present on the light reaching the main photo-detector.

While the speedmeter concept looks very promising from a theoretical point of view, an experimental demonstration is missing so far. There are currently efforts to set up a speedmeter proof of concept demonstration experiment [117].

## 4 Future sensitivity improvements

There is significant effort underway in order to install and commission the hardware that is necessary to realise the sensitivity improvement of the advanced detector network. It is planned that these detectors will come online in 2015 and reach full design sensitivity around 2019 for aLIGO and 2021 for AdV [37]. While this work is progressing it is essential to consider upgrade scenarios for the advanced detector network which could provide a further improvement in a factor of 3–5 in the strain sensitivity. As hardware upgrades typically take 10–15 years to develop from the lab and install into the kilometre scale detectors, there is a strong targeted programme of future R&D which is directed at upgrades and future detectors. In this paper, we will only discuss future upgrades to the advanced detector network. However, the reader is referred to the design study of the Einstein Telescope which focuses on a future 3rd generation detector in Europe. This instrument features underground operation, arm lengths of 10 km and the use of dual temperature detector operation to allow reduction of fundamental noise sources [69, 118].

### 4.1 Injection of squeezed light with a frequency dependent squeezing angle

For improving the aLIGO sensitivity initially in a certain frequency band and later over the full frequency range, the application of squeezing has been suggested [119, 120] since of all concepts for improving quantum noise beyond the SQL it has achieved the highest maturity.

While during the previous decade the focus of squeezed light research within the gravitational wave community was set on prototyping suitable squeezed light sources providing sufficient squeezing level (more than 12 dB) [121] as well as extending the range of squeezing to frequencies as low as 1 Hz [122], current research efforts concentrate the low-noise implementation of squeezed light in a full-scale advanced gravitational wave detector.

In order for squeezing to providing improved quantum noise at all frequencies, i.e. in the frequency range limited by shot noise as well as in the range limited by back-action noise, squeezed light states need to be injected, where the squeezing ellipse features a frequency dependent orientation [123]. Such a frequency dependent squeezing angle can be realised by using the dispersion in reflection of a detuned cavity, often referred to as filter cavity (see Figure 12).

The key difficulty in the realisation of such a filter cavity is to provide a very low bandwidth (of the order of the frequency cross over between shot and back-action noise) combined with extremely low optical losses in order to not destroy the squeezing [124].

**Figure 14.**
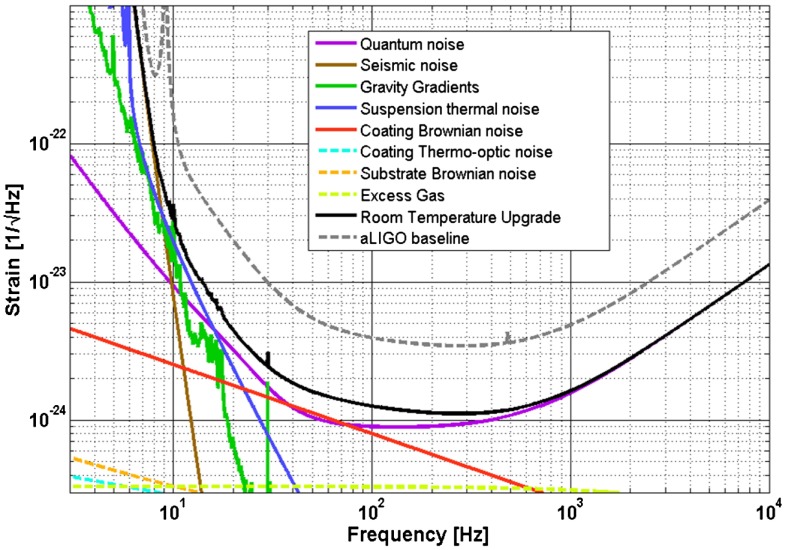
The strain sensitivity improvement for a room temperature upgrade based on fused silica [120]. (The colour version of this figure is included in the online version of the journal.)

The quantum noise curve for the aLIGO upgrade shown in Figure 14 assumes a 300 m long filter cavity with a roundtrip loss of 30 ppm, an input mirror transmission 425 ppm and a detuning frequency of -16.8 Hz [120]. In addition to further improve the sensitivity at the low frequency end, the weight of the test masses was assumed to be increased from 40 to 160 kg.

### 4.2 Warm upgrades

#### 4.2.1 Suspension thermal noise

As noted previously, lowering the suspension thermal noise requires the use of ultra low loss materials. Warm upgrades (e.g. room temperature operation) will likely require the use of heavier test masses (≃160 kg) to lower the effect of radiation pressure noise (see Equation (16)). Fortunately, fused silica is available up to diameters of approximately 55 cm which allows sufficiently heavy test masses to be fabricated. Suspending heavier test masses will require the fused silica fibres to be scaled in dimension such that the region of thermoelastic cancellation is equal to 1.3 mm (see Section 3.2 and Equation (6)). With current CO${}_{2}$ laser pulling technology this does not seem to be a challenging requirement. It is also advisable to use longer suspensions as this increases the ratio of the energy stored in gravity to that stored in elasticity. This has the effect of improving the dissipation dilution (D) and thus lowering the effective mechanical loss angle of the suspension. In aLIGO suspension lengths of up to 1.5 m can be accommodated with some re-working of the support structure necessary to hold the seismic isolation system. It should be noted that it is desirable to maintain the violin mode frequencies of the tensioned fused silica fibres in the region of 500 Hz and thus any length increase of the suspension will tend to reduce these frequencies. One possibility to further increase the frequency is to utilise thinner fibres which operate at a higher stress. In the advanced detectors, the fibres operate at about 16% of their ultimate tensile strength. Working at 30% would ensure that the violin modes remain at the same frequency [125]. An added benefit of both a longer final stage and thinner fibres is that the vertical mode of the suspension lowers from 9 Hz to 6.2 Hz, thus providing additional vertical isolation at 10 Hz (Equation (4)). Optimising the geometry of the neck of the fused silica fibres is also a technique to further improve the suspension thermal noise. By pulling from thicker (5 mm diameter) fused silica stock more of the energy can be stored in the thin section of the fibre which ultimately maintains a high dissipation dilution.

With such improvements to the fused silica final suspension stage, care must be taken to ensure that the performance is not limited by other components of the quadruple pendulum. Modelling has shown that the final stage of vertical cantilever springs, which are currently fabricated from maraging steel, will begin to limit the performance of the final pendulum stage if improvements at the level of 2–3 can be made in the pendulum thermal noise. One possible solution is to utilise fused silica springs in this final stage which will have a loss angle roughly $10^{-3}$ lower than the maraging steel [126]. Challenges which need to be overcome include (i) making springs sufficiently strong with tensile strength of up to 800 MPa, and (ii) providing protective overcoats such that the silica springs can be handled and clamped into the suspension. There is significant interest in the use of coatings such as Diamond Like Carbon (DLC) to provide robust protective layers.

#### 4.2.2 Coating thermal noise

For coating thermal noise, the heavier test mass (with diameter ≃55 cm) will allow a reduction of 1.6 in the coating thermal noise via larger beam diameter. Further improvement will be achieved through the combination of improved coatings and/or new materials. As mentioned previously, there is strong evidence that a first correlation between the mechanical loss angle and the doping of the Ion Beam Sputtered tantala coatings has been observed, and future work in this area aims to use this information to develop coatings with improved loss. There is also significant research into alternative high index of refraction materials including Hafnia, Zirconia, Niobia and silicon nitride [127]. Furthermore, the use of doping to further lower the mechanical loss, and stabilise the coatings under the application of higher annealing temperatures ($>600^{\circ }C$) may also lead to additional improvements. Any reduction of the high index material will also require a parallel approach to studying lower mechanical loss low index layers, which although are lower, cannot be neglected. The study of slow annealing processes which reduce the total stress in the coating layers appears to be a possible area of future R&D, in addition to doping in order to stabilise the coatings against crystallisation. Conservative assumptions suggest that a further factor of 2 in the loss angle may be achieved, giving a total gain of ≃3.2 when combined with larger beam diameter.

#### 4.2.3 Overall improvement from warm upgrades

With the improvements stated above, a warm upgrade could potentially offer a factor of 3 improvement in the strain sensitivity as shown in Figure 14. This would correspond to an improved event rate by a factor of 27 compared to the advanced detector network. Preliminary analysis [120] has shown that such an upgrade can be obtained for a fraction of the hardware cost of a new infrastructure, such as the Einstein Telescope.

**Figure 15.**
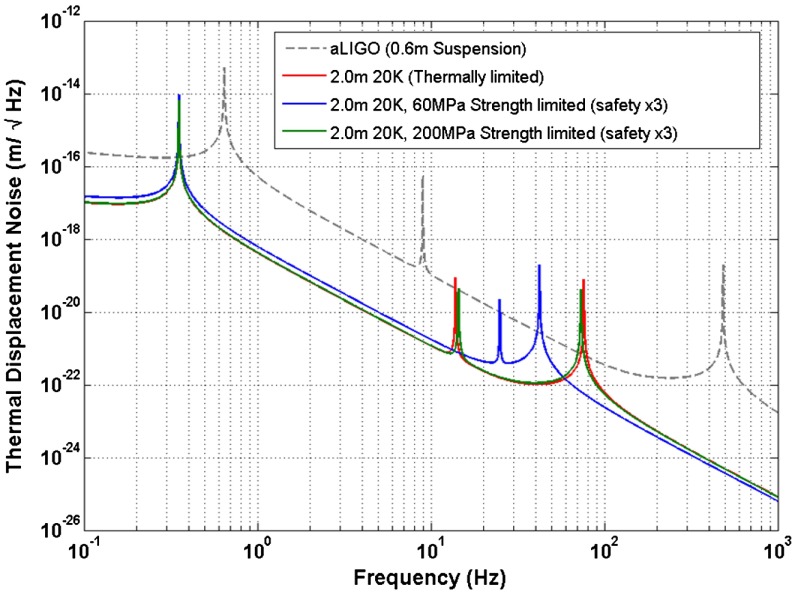
The suspension thermal noise performance for a variety of silicon suspensions which are both strength and thermally limited. (The colour version of this figure is included in the online version of the journal.)

### 4.3 Cryogenic upgrades

Cryogenic upgrades assume a combined approach of lowering the mechanical loss angle and the temperature to provide improved thermal noise performance [69, 126, 128]. While amorphous materials (e.g. fused silica) work extremely well at room temperature, a low temperature dissipation peak at around 40 K limits their use for cryogenic applications. Crystalline materials on the other hand, such as silicon and sapphire, do not show such broad dissipation peaks and are the best candidates [69, 129]. An additional benefit of crystalline materials is that they display high thermal conductivity in the region $>500\;Wm^{-1}K$ which is important for both reducing the problems associated with thermal lensing with high laser powers and extracting heat from the optic. Silicon is the baseline material for the European Einstein Telescope design study. A significant benefit is the fact that large sample sizes will become available within the next 10 years (e.g. >50 cm diameter for 200 kg optics) as a result of the semiconductor industry. However, further R&D is required to develop 1550 nm laser technology to allow transmissive interferometry. Sapphire is the material which is the baseline for the KAGRA detector currently being built in Japan. While the maximum diameter of the available sample, and thus the ultimate mass of the optic, is lower than silicon, a benefit is that sapphire can be operated at 1064 nm with transmissive optics.

One choice which is important to make is the operating temperature of the detector. Silicon exhibits a zero in its thermal expansion coefficient at both 120 and 18 K [69] and thus the thermoelastic noise contribution can be made negligible at this point (with remaining loss terms due to surface and bulk). Operating at 120 K allows significant heat (≃10 W) to be extracted via radiative cooling to a nearby cold structure [128] although the thermal noise performance is not necessarily as good as a low temperature operation (assuming identical laser power). On the other hand, operating at 18 K offers high gain in terms of thermal noise performance, but with the challenge of needing to extract several tens of milliwatts of heat along the suspension fibres. The deposited heat originates from the absorption of the incident laser beam onto the mirror coatings (ideally a few ppm absorption) and also optical absorption of the laser beam as it travels through the transmissive optic of the interferometer (a few ppm/cm).

#### 4.3.1 Suspension thermal noise

Significant modelling has been undertaken on the silicon suspension thermal noise for both 120 and 18 K operation [126, 130]. Similar to warm upgrades, long suspension elements with thick attachment ends to optimise the stored energy into the thin fibre are necessary. There has already been work to test fabrication techniques which typically fall into two categories. The first requires the use of a heated pedestal growth technique [131] to pull a crystal fibre out of a melt. Challenges involved with this techniques include maintaining temperature stabilisation of the molten silicon bath and ensuring that the resulting crystal is single axis and not polycrystalline. The second method assumes etching or machining the fibres out of wafers which results in rectangular geometry suspension elements. Challenges with this method include making strong Silicon fibres which retain their strength after etching or mechanical machining.

As stated above, fabricating crystalline suspensions requires a careful analysis of the strength and thermal requirements of the support elements, in addition to reproducible bonding techniques [132, 133]. Current measurements [130] of the tensile strength of silicon elements yield values in the region 200–500 MPa, although further work to improve this value is very likely. From these measurements, it is possible to show that a 200 kg test mass suspension would be currently limited by strength requirements. If silicon could be made sufficiently strong, then the suspension elements would be reduced in cross-sectional area, ultimately improving the suspension thermal noise via improved dissipation dilution. It is important to note that for low temperature operation (e.g. <50 K), where radiative cooling is not viable, there is a further limitation that the elements must remain of sufficient cross-section to remove the deposited heat. Figure 15 shows an example of the thermal noise performance of a silicon suspension comprising a 200 kg test mass and 2 m long suspension elements. For reference the aLIGO design is also shown. Three silicon suspensions are shown: a thermally limited suspension (red line), a strength limited suspension operating at 60 MPa with a safety factor of 3 (blue line) and a strength limited suspension operating at 180 MPa with a safety factor of 3 (green line). The peaks correspond to the pendulum mode, the vertical mode and only the first violin mode (other modes at harmonics have been omitted). There is significant improvement potential for such suspensions operating at cryogenic temperature, with up to a factor of 50 improvement in the thermal noise performance at 10 Hz. Operating at higher stress values pushes apart the vertical mode and violin mode which is desirable. However, assuming a maximum stress of 200 MPa (60 MPa with a safety factor of 3) shows that strength of the silicon is currently the driving parameter.

#### 4.3.2 Coating thermal noise

There are significant gains to be made from coating thermal noise improvements. Again the use of heavier test masses allows the possibility of increasing the beam size by a factor of 1.6. As noted above, amorphous coatings are not ideal at cryogenic temperatures due to the observed low temperature loss peak. Thus, alternative coatings are currently under development based on crystalline materials which are grown with Molecular Beam Epitaxy. Two such coatings technologies are based on gallium arsenide (GaAs) and gallium phosphide (AlGaP). The AlGaAs coatings, whose high/low index layers are achieved by varying the concentration of aluminium (Al${}_{0.92}$Ga${}_{0.08}$As/GaAs for low/high index respectively), have been studied in detail in opto-mechanical experiments [134]. These coatings offer losses which are at the level of $4\;\times \;10^{-6}$ and absorption less than 6 ppm. A challenge is that the coatings are grown on a gallium arsenide substrate and thus have to be lifted off onto either silicon or sapphire substrates. Such techniques are reasonably standard in the microfabrication industry and have already been used to put coatings onto crystalline substrates which have been temperature cycled to 10 K and lower. There is further possibility to scale these up to larger substrates, although additional R&D to put these coatings onto curved mirror substrates and test their mechanical loss on representative size samples is necessary. The GaP coatings, whose high/low index layers are also achieved by varying the concentration of aluminium (Al${}_{0.9}$Ga${}_{0.1}$P/GaP for low/high index respectively), are lattice matched to silicon and can be grown directly onto these substrates with minimum defects. An upper limit on the losses of these coatings is $<2\;\times \;10^{-4}$ although work is ongoing to further measure this relatively new coating technology. The optical absorption is still too high for current interferometers, although the possibility of annealing these coatings may lead to a possible reduction. There is also significant research ongoing to study resonant waveguide mirrors [135–137]. These devices work by resonantly coupling incident light into a periodically structured grating and have the benefit of removing all coating layers. Recent loss measurements suggest that at cryogenic temperatures, the loss can be an order of magnitude lower than that of the Ion Beam Sputtered coatings and absorption is at the level of ≃100ppm. Again a combined approach of alternative coating technologies and materials is likely to deliver gains of up to a factor of 5–10 in mechanical loss. The coating thermal noise varies with the square root of the mechanical loss, as detailed in Equation (12), and when combined with the increased beam diameter, a significant gain of 8–16 in coating thermal noise can be expected.

### 4.4 Dual detectors

**Figure 16.**
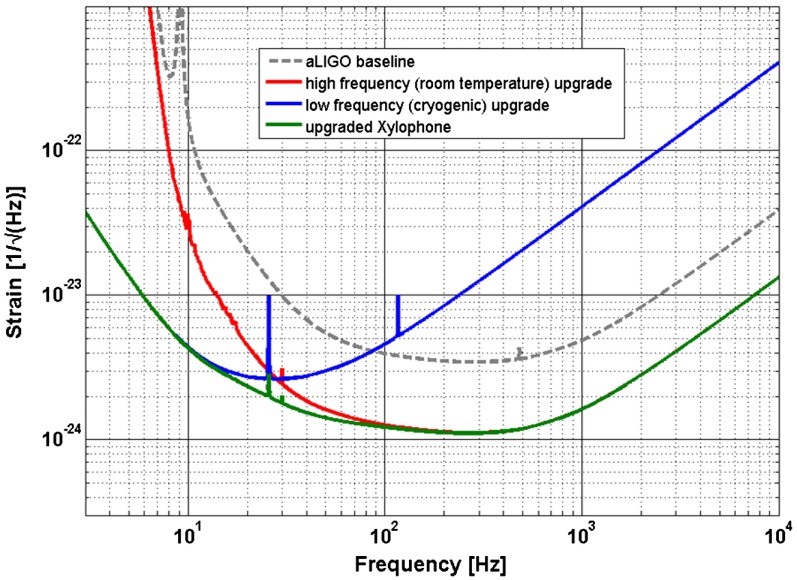
The strain sensitivity improvement for a cryogenic upgrade based on silicon at 20 K. Note that for the blue and green trace only thermal noise and quantum noise are considered. To achieve this level of sensitivity, methods have to be implemented to eliminate Newtonian noise and seismic noise correspondingly. (The colour version of this figure is included in the online version of the journal.)

There are challenges associated with operating a cryogenic detector at high laser power. Thus, an interesting concept includes the use of a dual operating scheme or Xylophone concept [138–140]. This would comprise a high frequency, room temperature detector, operating at high power and utilising fused silica as the baseline material. In parallel, a cryogenic detector, operating with low power and utilising crystalline silicon or sapphire, would form the low frequency detector. Such a configuration could provide the benefits of both operating schemes with broadband improvement. Figure 16 shows the strain sensitivity improvement for a Xylophone configuration. Only the fundamental noise sources are shown on this plot (quantum noise, suspension thermal noise, coating thermal noise) and thus such a detector would require a significant improvement or subtraction of the Newtonian noise background and seismic noise. When compared to aLIGO, the improvement in the strain sensitivity is ≃7 at frequencies less than 20 Hz.

### 4.5 Astrophysics

It is very likely that the first direct detections of gravitational waves will happen with the operation of aLIGO and AdV. However, following this vital milestone, there are many questions in astrophysics, fundamental physics and cosmology that can be addressed through further observations [12] in particular with the huge numbers of sources expected for ET [141]. At their design sensitivities aLIGO and AdV should have astrophysical reaches of a few hundred Mpc for coalescing compact binary sources. As mentioned in Section 1.3, it is estimated that they should expect to observe ∼40 coalescences of binary neutron stars per year [27] when they reach their design sensitivity. They could also potentially observe tens of binary stellar mass black hole systems and neutron star-black hole binaries, which are systems that have never been observed through electromagnetic observations. Whilst with ET thousands of these sources could be observed out to high cosmological redshifts [141]. Through their use as “standard sirens” [142] their distances can be determined and, provided a measure of the redshift can be estimated (see below), they can be used to determine cosmological parameters.

This also opens up the prospect of multimessenger astronomy in which gravitational wave and electromagnetic, or high energy neutrino/cosmic-ray, observations can be combined to get the most information about a source (see [143] for a review of multimessenger efforts with initial detectors, and [144] for how it could be used in the ET era). As gravitational wave detectors are sensitive to the whole sky, the localisation of short transient sources requires triangulation using signal arrival times in multiple detectors. With the two aLIGO instruments and AdV, it is expected that typically a source may be localised to tens of square degrees on the sky [37, 145]. This localisation would be further improved though the inclusion of KAGRA and/or the siting of one aLIGO instrument in India [146, 147]. Localising a gravitational wave source could allow it to be unambiguously associated with an electromagnetic transient, such as a γ-ray burst or supernova, providing unique insight into the mechanisms for these astrophysical events. Localising inspiral events could identify their host galaxies, allowing the redshift to be measured and enabling their use as cosmological probes.

There are many further exciting prospects that could arise from these observations, including probing neutron star equations of state and testing GR in extreme gravity situations. Many of these are described in more detail in e.g. [12, 141]. This is an extremely exciting time for the field of gravitational wave astrophysics with the advanced detectors coming online in 2015, first observations expected ≥2016 and a strong international programme of R&D for future upgrades and detectors.
